# Technical Data from Agfa

**Published:** 1989-05

**Authors:** 


### Technical Matters

**SPECIAL FEATURE ON BREAST IMAGING**
From AGFA.

It is well known that mammographic film should have dedicated processing. This is not only because it enables the sensitometric properties of the film to be matched more closely to the users requirements. It is also desirable because it enables the user to more easily attain the high standard of cleanliness needed for this exacting radiological examination. Furthermore, some processing machines are less likely to produce 'pinholes' than others.

Such a processing machine is the one fitted to the Curix Compact film handling and processing unit. Designed specifically for single emulsion films, the Curix Compact processor has a straight through film path, which means minimum roller contact.

The Curix Compact can be put on a long processing cycle and, with a choice of temperature, will produce ideal results. The Curix Compact can be chemically supplied from either a chemical mixer or by its own supply bottles.

When fitted with supply bottles and waste containers, no services are required, other than a 13 amp power point. This makes it a suitable daylight film handling and processing unit for isolated mammography departments or mobile mammography units where trailers are 30 foot or longer.

The cassettes used with the Curix Compact push the film to the chest wall edge when they are closed. Thus enabling very close body contact. These cassettes also have a unique magnetic contact system that will not change with use. Fitted with M R Detail screens they give excellent image detail without a high dose when used with Mamoray MR3 film.

For further information contact D.I.S. Division, Agfa-Gevaert Ltd., 27 Great West Road, Brentford, Middx., TW8 9AX.

